# Earliest fossils of giant-sized bony-toothed birds (Aves: Pelagornithidae) from the Eocene of Seymour Island, Antarctica

**DOI:** 10.1038/s41598-020-75248-6

**Published:** 2020-10-26

**Authors:** Peter A. Kloess, Ashley W. Poust, Thomas A. Stidham

**Affiliations:** 1 Department of Integrative Biology and Museum of Paleontology, University of California, Berkeley, CA 94720 USA; 2 Present Address: Department of Paleontology, San Diego Natural History Museum, San Diego, CA 92182 USA; 3 Key Laboratory of Vertebrate Evolution and Human Origins, Institute of Vertebrate Paleontology and Paleoanthropology, Chinese Academy of Sciences, Beijing, 100044 China; 4 CAS – Center for Excellence in Life and Paleoenvironment, Beijing, 100044 China; 5 University of Chinese Academy of Sciences, Beijing, 100049 China

**Keywords:** Palaeontology, Palaeoecology

## Abstract

While pelagornithid or ‘bony-toothed’ bird fossils representing multiple species are known from Antarctica, a new dentary fragment of a pelagornithid bird from the middle Eocene Submeseta Formation on Seymour Island, Antarctica represents a species with a body size on par with the largest known species in the clade. Measurements from the partial ‘toothed’ dentary point to a giant body size for the species, although the spacing among the pseudoteeth differs from that published for other pelagornithids. The discrepancy might suggest that previous techniques are not adequate for examination of incomplete material or that another factor such as phylogeny might impact size estimates and comparisons. Combined with a revised stratigraphic position in the early Eocene La Meseta Formation on Seymour Island for the largest pelagornithid tarsometatarsus known, these Antarctic fossils demonstrate the early evolution of giant body size in the clade (by ~ 50 Ma), and they likely represent not only the largest flying birds of the Eocene but also some of the largest volant birds that ever lived (with an estimated 5–6 m wingspan). Furthermore, the distribution of giant-sized pelagornithid fossils across more than 10 million years of Antarctic geological deposits points to a prolonged survival of giant-sized pelagornithids within the southern seas, and their success as a pelagic predatory component of marine and coastal ecosystems alongside early penguins.

## Introduction

Bony-toothed birds (Odontopterygiformes: Pelagornithidae) are an extinct clade of large, pelagic, volant birds with a fossil record spanning from the late Paleocene to the late Pliocene^1–3^ and a global distribution^4^. As their colloquial name suggests, the most obvious diagnostic characteristic of this clade is the modification of the tomial crest of the premaxillae, maxillae, and dentaries into a variety of tooth-like bony projections that lack dental tissues (or homology to teeth). The sizes and spacing of these projections vary across the clade but are consistent within species, following a set sequence of large and small pseudoteeth covered in life by the rhamphotheca^5,6^. This pseudodentition, along with hooked premaxillae and the presence of intraramal joints, has been hypothesized to indicate dietary preferences for fish or squid skimmed from the top of the water column^7,8^. Pelagornithids and the extinct teratorns (Teratornithidae) from the Neogene and Quaternary are the largest volant birds known, and while the body sizes of pelagornithids vary, the majority of known specimens and species derive from individuals considered large (3.5–4.5 m wingspan) and even giant (5–6 m wingspan)^9^.

**Figure 1:**
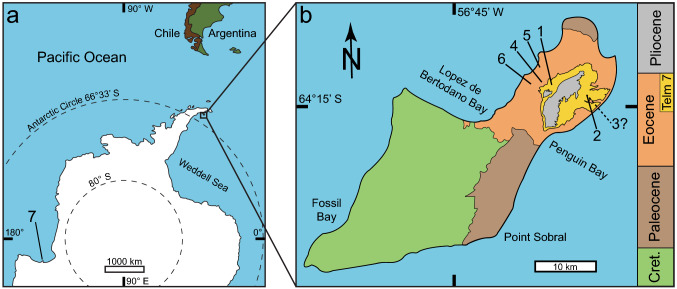
(**a**) Map of Antarctica with location of Seymour Island highlighted (modified from Google Earth, Image PGC/NASA). (**b**) Generalized geologic map of Seymour Island (modified from Montes et al.^13^). Numbers indicate localities containing Antarctic pelagornithid specimens: 1: UCMP RV8405 (present study); 2: DPV 13/84^61^; 3: Specimen MLP 78-X-26-1; 4: IAA 1/95^62^; 5: IAA 1/90^62^; 6: UCMP RV8702^47,63^ (present study); and **7**: Specimen USNM 494035^37,42^. Uncertainty in the placement of MLP 78-X-26-1 is explained in the text.

**Figure 2:**
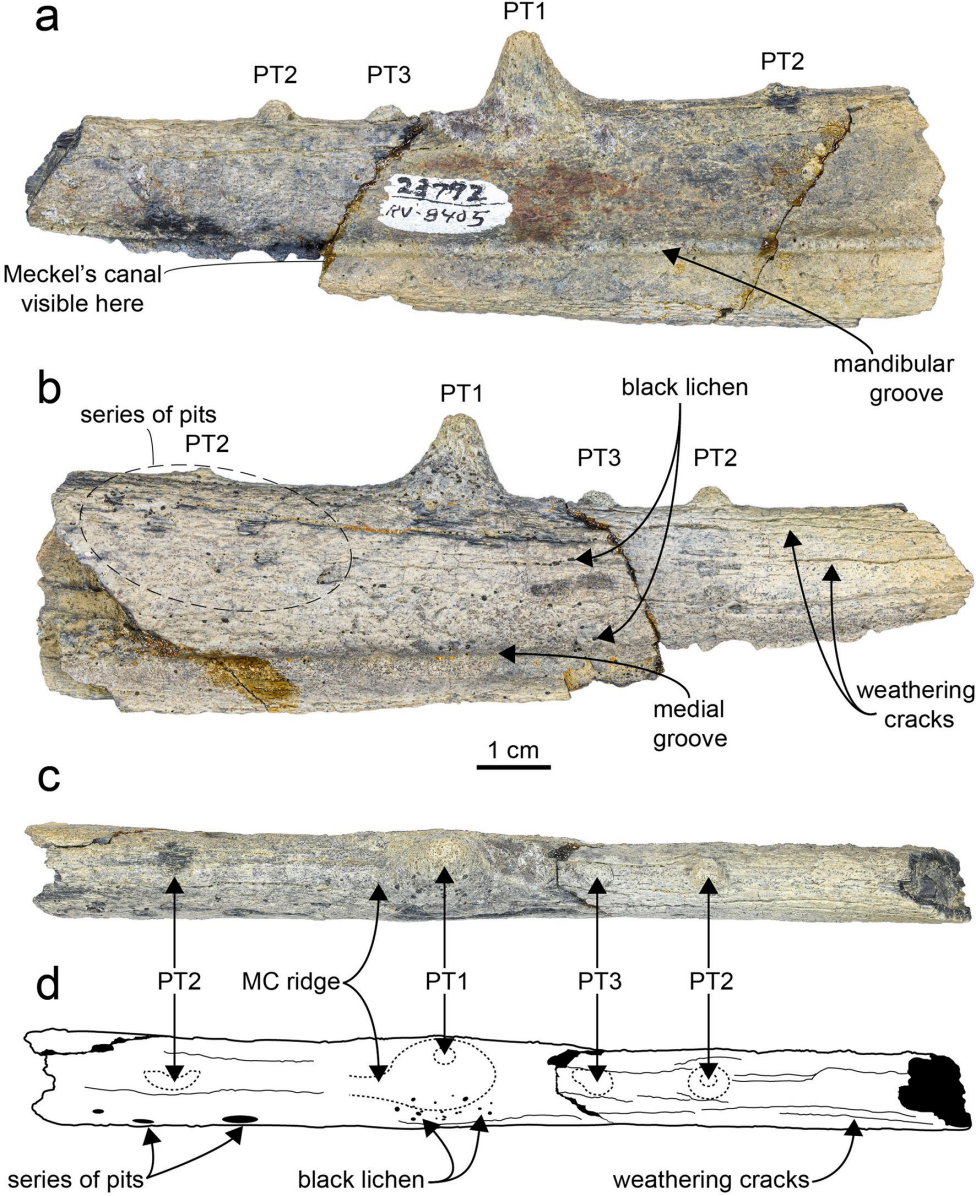
Left pelagornithid dentary fragment UCMP 323792 in lateral (**a**), medial (**b**), and dorsal views (**c**). (**d**) Line drawing of dorsal view to elucidate location of pseudoteeth. Pseudoteeth depicted as dashed outlines. Note on PT1 the presence of a mediocaudal crest and its tip is offset from the midline. *MC* mediocaudal, *PT* pseudotooth.

**Figure 3:**
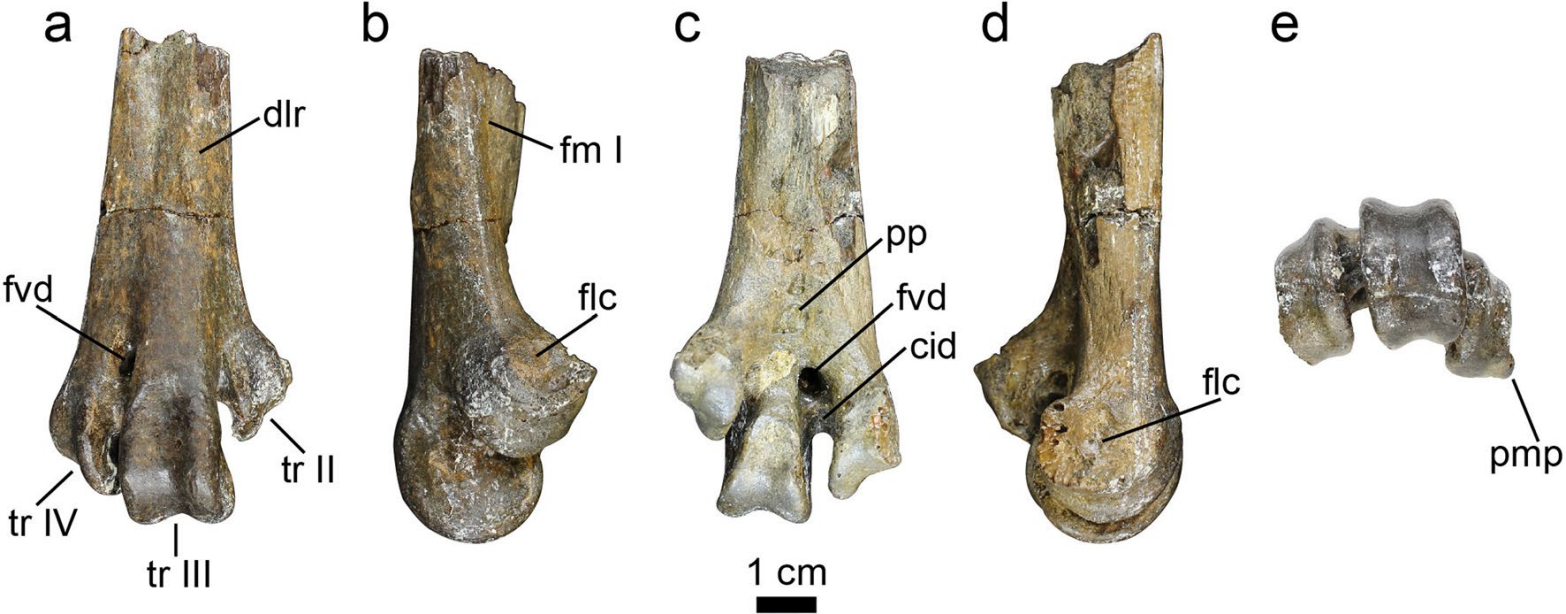
Photographs of the original fossil material of the pelagornithid distal right tarsometatarsus UCMP 322176 in: dorsal (**a**), medial (**b**), plantar (**c**), lateral (**d**), and distal views (**e**). Residual mold lines from the cast-making process can be observed on the medial, lateral, and trochlear surfaces (**b**,**d**, and **e**). Terminology from Baumel and Witmer^64^, Cenizo^45^, and Cenizo et al.^9^. Osteological abbreviations: *cid* canalis interosseus distalis, *dlr* dorsal longitudinal ridges, *flc* fovea ligamentum collateralium, *fm I* fossa metatarsi I, *fvd* foramen vasculare distale, *pmp* processus medianoplantaris, *pp* plantar “pit”, *tr* metatarsal trochlea.

Though they have a nearly global distribution, pelagornithid specimens from Antarctica are rare and limited to isolated elements; most of which derive from the Eocene sediments of the La Meseta and Submeseta Formations on Seymour (Marambio) Island, near the Antarctic Peninsula^9^ (Fig. 1). The published specimens from these formations include two partial maxillae, one fragment of a humerus, one dentary fragment, and one distal tarsometatarsus. We add to this assemblage by describing a > 12 cm long pelagornithid dentary fragment, University of California Museum of Paleontology (UCMP) 323792 (Fig. 2). We also revise the stratigraphic placement of the previously reported tarsometatarsus specimen, UCMP 322176, within the La Meseta Formation pelagornithid assemblage (Fig. 3). With these two specimens, the known pelagornithid record from Seymour Island is now represented by six specimens representing multiple taxa; three from the early Eocene (including the presence of a giant-bodied specimen in a temporal period previously represented only by large-sized individuals) and three from the middle to late Eocene (represented by giant-sized individuals). A nearly complete pelagornithid humerus awaits formal description and would add to this collection of specimens^10^.

## Geologic and paleontological background

**Figure 4:**
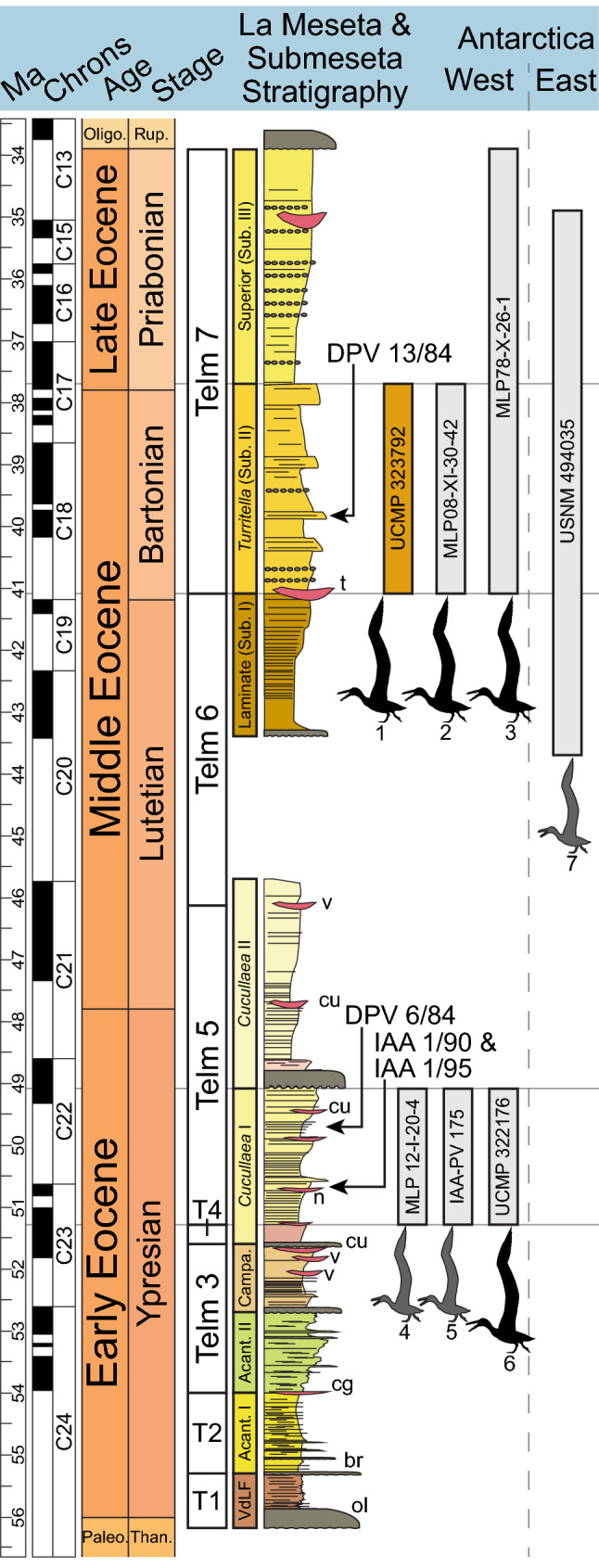
Geochronological context and estimated body size classes of known pelagornithid specimens from Antarctica. Left, stratigraphic section of the La Meseta and Submeseta Formations (modified from Beamud et al.^18^ and Montes et al.^19^) and pelagornithid specimens recovered from Seymour Island. Furthest right, the pelagornithid specimen found in East Antarctica (USNM 494035). Two methods (middle columns) are shown for the subdivision of the La Meseta and Submeseta strata. Numbers and pelagornithid outlines correspond to specimen localities displayed in Figure 1. Pelagornithid outlines are modified from Boessenecker and Smith^3^; the colors and relative sizes of these outlines correspond to the “large” (in gray) and “giant” (in black) size-types of Cenizo et al.^9^.

The geology of Seymour Island records Cretaceous to latest Eocene marine strata along with Pliocene to Pleistocene glaciomarine deposits restricted to the northern portion of the island^11–13^ (Fig. 1). These Eocene strata contain deltaic, estuarine, and shallow marine deposits filling an incised valley^14,15^, and the Eocene sediments have been subdivided using two different approaches. The first method designates “Telm” units (an acronym for Tertiary Eocene La Meseta) based on lithofacies^16^, and the second relies on unconformities to divide the La Meseta Formation into allomembers^17^. Some authors (e.g. Refs.^18,19^) have gone further in elevating the uppermost Submeseta Allomember to formation status and subdividing that new formation into the Laminate, *Turritella*, and Superior Allomembers (formerly the Submeseta I, II, and III units, respectively, of Montes et al.^13^). Here, we use both the Telm units and the allomembers in accordance with the work of various authors, and refer the reader to the combined stratigraphic column (Fig. 4) for correlations.

Recently, there has been uncertainty regarding the age of the La Meseta and Submeseta Formations. A combination of age-dating methods, such as strontium isotopes, magnetostratigraphy, and lithostratigraphy, indicate that the La Meseta Formation is early to middle Eocene in age and the Submeseta Formation is middle to late Eocene in age^18,19^. However, studies of dinoflagellate cysts support a middle to late Eocene age for the La Meseta Formation^20,21^. We refer to the dates generated from the combined methodology^19^ in Fig. 4 and throughout the text because of the inclusion of the Submeseta Formation into these geochronological and stratigraphic studies.

The La Meseta and Submeseta Formations have yielded many avian fossils, including penguins, falconiforms, procellariiforms, anseriforms, paleognaths, and pelagornithids^22^. The pelagornithids from these deposits are represented by six published specimens (Fig. 4). One partial maxilla: MLP 08-XI-30-42 was recovered from locality DPV 13/84^23^. This locality has been assigned to multiple, parallel subunits of the La Meseta Formation: within Telm 7^24^; at the base of level 38 sensu Montes et al.^13,25^; and within the *Turritella* Allomember (equivalent to the Submeseta II Allomember used by Acosta Hospitaleche et al.^26^). The *Turritella* Allomember has been dated between 41.1 and 37.7 Ma on Seymour Island^18,19^.

Another maxillary fragment (MLP 78-X-26-1) was described first by Tonni and Cione^27^ and further detailed by Tonni^28^. These authors placed the specimen within the upper portion of the La Mesesta Formation, though its location was not shown on their included maps. A published catalogue of La Meseta fossil material housed in the Museo de La Plata^29^ confirms that the specimen comes from an unknown locality and the authors attribute it to the highest stratigraphic unit, Telm 7. MLP 78-X-26-1 was later assigned to locality DPV 13/84 within the Submeseta II Allomember^9^ but it is unclear how the assignment to this locality was determined. We use the conservative placement of MLP 78-X-26-1 within Telm 7, which encompasses the *Turritella* and Superior Allomembers (formerly Submeseta II and III, respectively), to indicate this specimen’s stratigraphic placement (Fig. 4). Telm 7 has been dated between 41.1 and 34.0 Ma^12,13,18^.

A distal pelagornithid humerus (MLP 12-I-20-4) was recovered from locality IAA 1/95 (Fig. 4). Vizcaino and coauthors^30^ indicate that this pelagornithid-bearing locality is in the same stratigraphic horizon as the mammal-bearing locality IAA 1/90, located within a naticid gastropod-dominated conglomerate layer in Telm 5 and the *Cucullaea* I Allomember^31–33^. Additionally, a recently published rostral dentary fragment (IAA-PV 175) from locality IAA 1/90 was described by Acosta Hospitaleche and Reguero^34^. The *Cucullaea* I Allomember is dated between 51.6 and 49.1 Ma and Telm 5 is dated between 51.3 and 46.2 Ma^18,19^. These dates yield an estimate of 51.3–49.1 Ma for the age of localities IAA 1/95 and IAA 1/90.

A nearly complete pelagornithid humerus (SGO.PV 22001) was recovered during a 2011 expedition to Seymour Island and has been described as larger than *Pelagornis chilensis* with a morphology similar to other *Pelagornis* taxa^10^. However, this specimen is awaiting formal publication and as such, we have not included its exact stratigraphic and geographic placement in our figures. If the preliminary stratigraphic position of the humerus^10^ is accurate, then the stratigraphic distribution of specimens displayed in Fig. 4 with the conservative placement of MLP 78-X-26-1 also in Telm 7 would be reinforced.

Three fossil specimens from Seymour Island were identified originally as Pelagornithidae but have since been reassigned to other taxa. In a review of Seymour Island pelagornithid material, Cenizo et al.^9^ compiled known pelagornithid occurrences and reassigned two mandibular specimens previously identified as Pelagornithidae to non-pelagornithid taxa; MLP 83-V-30-1 has been identified as penguin and MLP 83-V-30-2 has been identified as fish. Recently, a partial dentary (IAA-PV 823) published initially as a pelagornithid^34^ was reidentified as a perciform fish^35^.

The only Antarctic pelagornithid specimen to date not found on Seymour Island is a fragmentary humerus (USNM 494035) collected from the McMurdo Erratics (erratic E303) at Mount Discovery, East Antarctica^36,37^. Based on the presence of molluscs^38^, terrestrial and marine microflora^39,40^, diatoms^41^, and siliceous microfossils^42^, an early middle to late Eocene age (43.7–34.9 Ma) has been determined for these glacial erratics (Fig. 4). Depending on the geochronological methods used, the humeral fragment is of a similar age to the Submeseta specimens (the combined methodology of Montes et al.^19^) or the La Meseta specimens (following Amenábar et al.^21^).

## Revised stratigraphic provenience for UCMP 322176

UCMP 322176 (formerly RV 22176; Fig. 3) represents the largest pelagornithid tarsometatarsus known. An expedition team from the University of California (UC) at Riverside collected the specimen during the 1986–1987 field season from locality UCMP RV8702 (formerly RV-8702) on Seymour Island. In 2003, the vertebrate paleontology collection from UC Riverside, including UCMP 322176, was transferred to the UCMP where it currently resides. During this collections transfer, specimen and locality numbers were converted from the UC Riverside (RV) system to conform with the UCMP database; for example, specimen RV 22176 is now recorded as UCMP specimen 322176, and locality RV-8702 is equivalent to UCMP locality RV8702.

The distal right tarsometatarsus (UCMP 322176) was described initially as belonging to Phrorusrhacidae^43^ with Tambussi and Acosta Hospitaleche^44^ confirming that identification and publishing the first image of this specimen (however, without identifying its specimen number). In a review of the phorusrhacid material from the Cretaceous and Paleogene of Antarctica, Cenizo^45^ reassigned the tarsometatarsus (identifying the specimen number as “UCR 22176”) to the Pelagornithidae. Tambussi and Degrange^23^ incorrectly refer to this specimen as cast UCR 22175; the specimen number used (revised as UCMP 322175), corresponds to a fragment of a premaxilla reassigned from Phorusrhacidae to an unknown genus and species of paleognath^45^.

UCMP 322176 has been attributed to the Submeseta Formation^9,45^. However, a review of the original locality information indicates that rather than the Submeseta Formation, the tarsometatarsus was recovered from the same stratigraphic horizon as UCMP locality RV8200 (formerly RV-8200) within Telm 5 (the La Meseta Formation). UCMP locality RV8200 is equivalent to the locality DPV 6/84^46^, stratigraphically higher than IAA 1/90^31,47^, and within the *Cucullaea* I Allomember^32^. The *Cucullaea* I Allomember is dated between 51.6 and 49.1 Ma, and Telm 5 is dated between 51.3 and 46.2 Ma^13,18^. These dates yield an estimate of 51.3–49.1 Ma for the age of fossils from UCMP locality RV8702 (Fig. 4).

For the first time, we include high resolution images of the original tarsometatarsus fossil to highlight aspects of its morphology (Fig. 3) because previous authors have published only images of casts made from UCMP 322176^9,44,45^. A detailed description of this tarsometatarsus, including observations of characters that this specimen shares with the *Dasornis* and *Pelagornis* morphotypes of Bourdon et al.^48^ and size comparison to other known distal tarsometatarsus fragments, was presented by Cenizo^45^, and the specimen was assigned further to a “giant” size-type (estimated 5–6 m wingspan) by Cenizo et al.^9^. The reassessed stratigraphic placement of this specimen to the La Meseta Formation (this study), where it joins other La Meseta specimens identified as “large” size-types (estimated 3.5–4.5 m wingspan sensu Cenizo et al.^9^), indicates that Seymour Island was inhabited by two different size classes of pelagornithids during the early Eocene.

## Materials and methods

Here, we describe a previously unpublished partial pelagornithid dentary (UCMP 323792) collected by a team from UC Riverside in 1983 during an expedition to Seymour Island, Antarctica (Fig. 1). The pelagornithid dentary was discovered at a site (UCMP RV8405) in the highest stratigraphic unit (Telm 7) of the middle to late Eocene Submeseta Formation (Fig. 4). According to the original field notes, the locality sits atop the most basal resistant sandstone bed of Telm 7, which overlies the unconformity of Telm 6. This description places the pelagornithid-bearing locality within the lower *Turritella* Allomember which has been dated between 41.1 and 37.7 Ma using lithostratigraphic, isotopic, and magnetostratigraphic data^13,18^.

**Figure 5:**
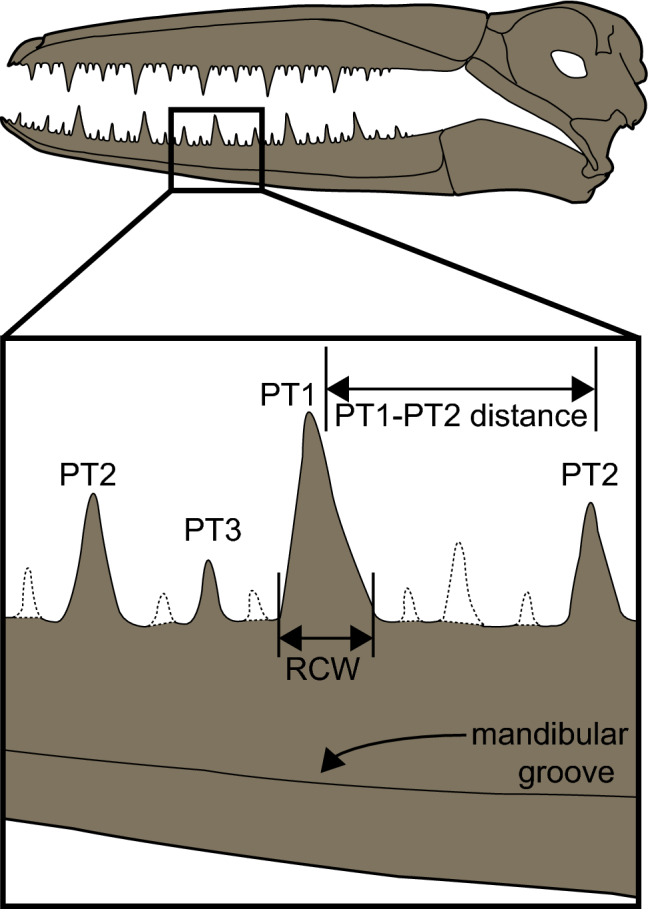
Diagram approximating the location of the dentary fragment, UCMP 323792, within a pelagornithid mandible (modified from Louchart et al.^5^). PT numbers indicate size class of pseudotooth, with PT1 representing the largest “teeth” and PT3 the smallest “teeth.” Bars indicate method for measuring rostrocaudal width (RCW) and the distance between PT1 and PT2s. Dashed lines indicate the possible locations of missing pseudoteeth. *PT* pseudotooth, *RCW* rostrocaudal width.

For analysis of the tooth-like projections on UCMP 323792, we follow Louchart et al.^5^ in classifying the pseudoteeth by size (Fig. 5). PT1 corresponds to the widest (at the base) pseudotooth class and PT3 to the smallest. Base width of the pseudoteeth was measured following Louchart et al.^6^ with the following modifications based on the preservation of UCMP 323792: (1) Given that only one PT1 is preserved, to calculate the intervening space between PT1s, we measured the space between the PT2s present; and (2) Given that two PT2s are preserved, we measured the rostrocaudal width of each and calculated the ratios based on each of these pseudoteeth.

We follow Mayr et al.^49^ with regard to the taxonomy of Neogene pelagornithid species which have all been assigned to *Pelagornis*.

## Description

### Dentary body

*Lateral aspect*—UCMP 323792 is a partial left dentary 12.2 cm long, preserving a region of the mandible rostral to the intraramal joint (Fig. 2). The dentary is relatively flat with a prominent mandibular groove (lateral longitudinal sulcus of Harrison and Walker^50^; external groove of Stidham^51^; neurovascular sulcus of Mayr and Rubilar-Rogers^52^; longitudinal furrow of Mayr et al.^53^) tracing the ventral length of the specimen. The groove is asymmetric, with a flatter ventral side compared to the gently sloping dorsal side. At the caudal end of the fragment, the groove is 11.4 mm dorsal to the ventral edge of the dentary, and 9.0 mm dorsal to that edge at the rostral end. The groove is 19.5 mm ventral to the dorsal edge of the dentary at the caudal end, and 15.8 mm ventral to the dorsal edge of the rostral preserved end. Overall, the groove approaches the ventral edge of the bone rostrally.

Though the rostroventral portion of the dentary is missing, the dorsal edge of the mandibular groove and Meckel’s canal are visible. This specimen shows that the groove is immediately adjacent to Meckel’s canal and thus likely related to the nutrient supply for the growing multipart rhamphotheca.

Given that UCMP 323792 derives from an inexact location within the dentary rostral to the intraramal joint, we compared measurements of its dorsoventral height to those collected from published images of complete dentaries from the largest pelagornithids, *P. chilensis*^52^ and *P. sandersi*^54^, both “giant” size-type pelagornithids^9^ from the Miocene and late Oligocene, respectively. Measurements from these specimens were collected from the base of the most rostral and most caudal PT1s, as well as the PT1 closest to the midpoint between them (Table 1). The rostral height measurement from UCMP 323792 (24.8 mm) is greater than the most rostral heights of *P. chilensis* and *P. sandersi*, 19.9 and 9.0 mm respectively, and just less than heights from their midpoints, 28.7 and 25.5 mm respectively. The most caudal heights of these species (*P. chilensis*: 40.1 mm; *P. sandersi*: 34.2 mm) is greater than the caudal height of UCMP 323792 (30.9 mm). Based on these measurements, UCMP 323792 falls well within the range of heights of these largest known pelagornithids and the fragment likely comes from near the rostrocaudal midpoint of the dentary.

**Table 1:** Dorsoventral height measurements (in mm) from large complete pelagornithid dentaries.

Species	PT1 (most rostral)	Approximate middle PT1	PT1 (most caudal)
*Pelagornis chilensis*	19.9	28.7	40.1
*Pelagornis sandersi*	9.0	25.5	34.2

Measurements were collected from published images at the locations of pseudoteeth but do not include PTs in these height measurements. Columns are arranged rostral (left) to caudal (right).

While UCMP 323792 has low, worn pseudoteeth similar to the oldest, smallest, and geographically closest pelagornithid to Antarctica, *Protodontopteryx ruthae*^53^, the preserved pseudoteeth in the maxilla and mandible of *Pr. ruthae* from the early Paleocene of New Zealand^53^ are weathered to the point that size class identification is impossible though it can be noted that the specimen exhibits approximately regular spacing of its pseudoteeth. The entire length of the preserved right dentary of *Pr. ruthae* measures less than the preserved length of UCMP 323792, and helps to document the very large diversity of body sizes within the clade.

*Medial aspect*—A wide shallow groove is visible along the ventral dentary. Mayr and Rubilar-Rogers^52^ used the term “neurovascular sulcus” for both the medial and lateral mandibular grooves in their figures of *P. chilensis*. The portion of the dentary dorsal to the groove is convex in profile and dorsoventrally taller (16.4 mm rostrally and 24.9 mm caudally) relative to the ventral dentary edge (~ 6.3 mm). Evidence of erosive events (pits, black lichen, and weathering cracks) are readily visible on this side of the specimen. Similar evidence of wear can be observed on the rounded and broken pseudoteeth. Lichen and related pits, resulting from apothecia, have been observed on fossils from the Submeseta Formation^55^ and their presence indicates the dentary had been exposed medial side up at the subaerial surface prior to its discovery^56^.

### Pseudoteeth

Four low, worn pseudoteeth are visible (Fig. 2). All of the pseudoteeth exhibit the remnants of a mediocaudal ridge, similar to those observed in other pelagornithids^57^. Based on the regular pattern of pseudotooth spacing observed in other pelagornithids, some of the smaller bony projections may have been worn away from UCMP 323792, and the possibility that even smaller, intermediately-spaced pseudoteeth (i.e. PT4s and PT5s) were present previously and also worn away cannot be ruled out (Fig. 5). It is likely that there is a pseudotooth missing between the PT1 and the caudalmost PT2, and one missing rostral to the most rostral PT2. Measurements from UCMP 323792, including rostrocaudal pseudotooth width taken at the base (where pseudotooth meets the dentary), height from pseudotooth base to apex as preserved, and distance along the dorsal surface of the mandible to the next caudal pseudotooth, are presented in Table 2.

**Table 2:** Measurements (in mm) collected from the partial pelagornithid dentary, UCMP 323792.

	PT2 (rostral)	PT3	PT1	PT2 (caudal)
Width at base	4.8	4.9	14.7	5.8
Height	2.6	2.1	10.5	1.4
Distance to next caudal PT	14.5	18.2	33.7	NA

Columns are arranged rostral (left) to caudal (right) in order of pseudoteeth preserved.

The distance measured from the existing PT1 to each of the remaining PT2s is greater than similar measurements from the largest pelagornithids, *P. chilensis* and *P. sandersi*. Since there is only one PT1 present in UCMP 323792, we measured the distance between the PT2s present as a proxy for calculating the space intervening between PT1s, and estimate that distance as 66.5 mm. This estimate of pseudoteeth spacing from UCMP 323792 is greater than all measurements compiled from various pelagornithids by Louchart et al.^6^, except those within the larger range of measurements from *P. chilensis* (ranging from 53.9 to 76.0 mm). The base width of the PT1 present (14.7 mm) also is larger than those compiled by Louchart et al.^6^, except for the larger measurements from *P. chilensis* (12.5–15.6 mm) and *P. longirostris* (13.3–15.9 mm), a pelagornithid of unknown Cenozoic age^52^ with cranial dimensions similar to *P. chilensis*^9^. Based on these measurements, UCMP 323792 may represent one of the largest pelagornithids found to date.

### Pseudoteeth length and spacing

The first reconstruction of a pelagornithid rostrum from *Dasornis* (*Odontopteryx*) *toliapica* indicates a repeating pattern of pseudoteeth^58^. Howard’s^59^ description of *Pelagornis* (*Osteodontornis*) *orri* provided details of the pattern of spacing and placement of pseudoteeth sizes; the largest pseudoteeth were spaced regularly along the length of the rostrum with the interstitial space bisected by moderately-sized pseudoteeth and then further split evenly by the presence of the smallest pseudoteeth. Although this pattern of pseudoteeth spacing has been observed in pelagornithid specimens identified since Howard’s^59^ observations, exceptions have been noted, for example at the tip of the rostrum^51^ or duplicate PT2s between PT1s^60^.

**Figure 6:**
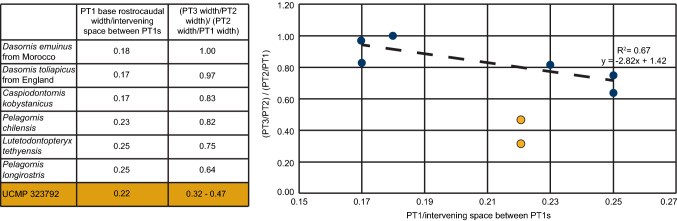
Measurements and regression of pseudoteeth data in pelagornithids. Measurements taken from UCMP 323792 and published pelagornithid specimens (Louchart et al.^6^). On the right side is a graphical plot of these measurements (published specimens in blue and UCMP 323792 in gold) with the associated regression line. Entries for UCMP 323792 show a range of numbers because measurements were taken from both PT2s present. Since only one PT1 remains, the “intervening space between PT1s” was calculated by measuring the space between the PT2s present.

Louchart et al.^6^ report known differences in the distribution and size of pseudoteeth between odontopterygiform species and calculated a regression line, based on the space between the largest pseudoteeth (PT1s) and a ratio of the rostrocaudal widths of pseudoteeth of different sizes, that supports a proposed mechanism for pseudotooth size and spacing based on inhibition zones. UCMP 323792 does not follow the pattern described by Louchart et al.^6^, but rather maintains a relatively low (extrapolated) intervening space between PT1s, as well as a lower value for the ratio of pseudotooth widths (Fig. 6). To account for the difference between the reported regression line^6^ and calculations from UCMP 323792, we consider taphonomic effects which have altered the specimen including, but not limited to, weathering of the pseudoteeth diminishing in vivo base widths and the loss of PT1s. Alternatively, measurements from UCMP 323792 may not align with the regression line for biological reasons, such as: this specimen may reflect a different ontogenetic stage or clade of pelagornithid from those specimens used in previous calculations, and thus may have a different pattern of pseudotooth spacing altogether. However, the regression line as published does not prescribe identification to taxon or ontogenetic age based on pseudotooth measurements and would require the addition of more specimens to properly make these assessments.

## Discussion

### Dentary size regression

The new pelagornithid dentary fragment described above (UCMP 323792) provides an opportunity to reexamine the previously identified Antarctic pelagornithid fossils, offering insight into the presence of coexisting morphotypes in the Southern Ocean throughout the Eocene. The measured and extrapolated spacing between pseudoteeth of UCMP 323792 point to a giant individual, but the use of widths from worn pseudoteeth may not be well-suited for detailed comparisons. Most rostral specimens of pelagornithids are partial, fragmentary, or taphonomically altered. If the metric of Louchart et al.^6^ is inappropriate for incomplete specimens, such as UCMP 323792, the general utility of many specimens for size regression is called into question, as is the use of spacing for taxonomic discrimination except in cases where intact segments can be confidently positioned within the oral cavity. The regression calculated by Louchart et al.^6^ also excluded specimens with the smallest pseudoteeth (PT4s and PT5s)—those most susceptible to erosion and weathering—and thus potentially excluded exceptionally preserved specimens.

### Early occurrence of “giant” pelagornithids in Antarctica

The updated stratigraphic context for the pelagornithid distal right tarsometatarsus (UCMP 322176) implies the presence of a “giant” pelagornithid taxon in the early Eocene of Antarctica. The other pelagornithid material from the early Eocene La Meseta Formation (a distal humerus, MLP 12-I-20-4, and a partial dentary fragment, IAA-PV 175) exhibit morphology and size similar to “large” pelagornithids, such as cf. *Gigantornis* sp.^9^. The stratigraphically-reassigned tarsometatarsus (UCMP 322176) possesses morphological affinities to both the *Dasornis* and *Pelagornis* morphotypes^45,48^ though its width is greater than tarsometatarsi identified as “giant” *Pelagornis* taxa^9^. The difference in sizes of these specimens suggests that “large-” and “giant-sized” pelagornithid taxa co-occurred in the early Eocene of Antarctica, and that the giant size class of pelagornithids evolved quite early in their history.

With the reassignment of a recently published partial dentary (IAA-PV 823^34^) as a perciform fish, the late Eocene of Seymour Island is currently unambiguously represented by only “giant” size-type specimens of pelagornithids. However, the fragmentary humerus (USNM 494035^36,37^) from Mount Discovery indicates the presence of multiple pelagornithid size-types and taxa across Antarctica during this time. Therefore, it would appear that the two pelagornithid morphotypes of Bourdon et al.^48^ and the largest two pelagornithid size-types of Cenizo et al.^9^ spanned from the early Eocene to the late Eocene of Antarctica. The Eocene La Meseta and Submeseta pelagornithid specimens suggest the presence of an unnamed species larger than known Eocene taxa. Known specimens that approach the size of these Antarctic specimens have been recovered from Oligocene and Miocene strata, but not yet the Eocene. Furthermore, the reassigned La Meseta tarsometatarsus (UCMP 322176), with characteristics intermediate between the two accepted morphotypes^48^, may represent an unnamed species larger than all known pelagornithid taxa. None of the specimens from Antarctica have been identified to genus, nor have any of them been used to establish new taxonomic names. However, there are likely at least two taxa (or species lineages) present through the Eocene of Seymour Island, and that only with the discovery and description of more overlapping skeletal elements may we begin to evaluate the alpha level diversity of pelagornithids present in this ancient ecosystem. Nevertheless, these unnamed remains are a tantalizing suggestion that the largest bird that ever flew may have soared its way over the Antarctic seas during an Eocene with a unique, and distinctively large-bodied, coastal avifauna. In addition, the distribution of pelagornithid body sizes in the same pelagic Antarctic ecosystem likely reflects ecological differences related to diet or foraging strategy, and indicates stability in those ecological niches through much of the Eocene. This updated fossil record of pelagornithids on Seymour Island reinforces the ideas that along with penguins and paleognaths^22^, pelagornithids were a common and even a dominant avian clade throughout the Eocene of Antarctica, and potentially competed with other soaring birds for foraging and nesting spaces. These pelagornithids would have occupied a high trophic level in Antarctic seas, a role today filled by albatrosses and other pelagic avian clades, and the combined utilization of marine resources by pelagic birds and penguins seen today likely extended into the deep past.

## Abbreviations

IAA: Instituto Antártico Argentino, Buenos Aires, Argentina
DPV: Departamento Paleontología Vertebrados, Museo de La Plata, Buenos Aires, Argentina
MLP: Museo de La Plata, Buenos Aires, Argentina
PT: Pseudotooth
RV: University of California, Riverside Vertebrate Paleontology collections (currently housed at the UCMP)
Telm: Tertiary Eocene La Mesesta
UCMP: University of California Museum of Paleontology, Berkeley, California, U.S.A.
USNM: National Museum of Natural History Paleobiology collection, Washington D.C., U.S.A
